# Microbiome transplants may not improve health and longevity in *Drosophila melanogaster*

**DOI:** 10.1242/bio.061745

**Published:** 2025-01-21

**Authors:** Benjamin H. Levine, Jessica M. Hoffman

**Affiliations:** Department of Biological Sciences, Augusta University, Augusta, GA 30912, USA

**Keywords:** Aging, Longevity, Microbiome, Health, Metagenomics, Metabolomics

## Abstract

The gut microbiome, which is composed of bacteria, viruses, and fungi, and is involved in multiple essential physiological processes, changes measurably as a person ages, and can be associated with negative health outcomes. Microbiome transplants have been proposed as a method to improve gut function and reduce or reverse multiple disorders, including age-related diseases. Here, we take advantage of the laboratory model organism, *Drosophila melanogaster*, to test the effects of transplanting the microbiome of a young fly into middle-aged flies, across multiple genetic backgrounds and both sexes, to test whether age-related lifespan could be increased, and late-life physical health declines mitigated. Our results suggest that, overall, microbiome transplants do not improve longevity and may even be detrimental in flies, and the health effects of microbiome transplants were minor, but sex- and genotype-dependent. This discovery supports previous evidence that axenic flies, those with no gut microbiome, live healthier and longer lives than their non-axenic counterparts. The results of this study suggest that, at least for fruit flies, microbiome transplants may not be a viable intervention to improve health and longevity, though more research is still warranted.

## Teaser

**Summary:** Microbiome transplants have been proposed as a method to mitigate age-related health declines. Here we determine that microbiome transplants generally fail to extend lifespan and improve health in *Drosophila melanogaster*.

## INTRODUCTION

Average longevities are increasing across the globe; however, this is leaving many individuals to spend years, potentially decades, in poor health (Sole-Auro and Alcaniz, 2015). Therefore, there is an imperative need to discover interventions that can improve the health of older adults. Biomedical researchers have been focusing on the molecular mechanisms that drive these negative late-life outcomes, often termed the hallmarks of aging (López-Otin et al., 2023). One recent addition to the hallmarks of aging and a potential avenue for intervention to improve late-age health is mitigating dysbiosis of the gut. Dysbiosis refers to imbalances in the composition and concentration of the gut microbiome that typically lead to negative health outcomes (DeGruttola et al., 2016).

The microbiome represents all the bacteria, viruses, fungi, archaea, and eukaryotes that inhabit an organism's body and can have differing impacts on its hosts (Ferranti et al., 2014). The gut microbiome in *Homo sapiens* is involved in a number of beneficial functions, ranging from digestion and immunity to neurological function (Gwak and Chang, 2021). However, if dysbiosis occurs, these same microorganisms can have detrimental effects, including chronic conditions (e.g. inflammatory bowel disease, obesity, cancer, and ulcerative colitis), as well as acute infectious diseases (Ono et al., 2008; Zhang et al., 2015). Dysbiosis can be caused by multiple intrinsic and extrinsic factors, including the host's genetic background, diet, overall health, lifestyle, environment, and, interestingly, age (Hrncir, 2022).

One potential method to correct dysbiosis of the gut is using a fecal microbiota transplant (FMT), which replaces the unhealthy microbiome of a patient with a healthy donor microbiome (Borody and Campbell, 2011). FMTs have been used in modern medicine to treat gastrointestinal specific diseases, including ulcerative colitis, constipation, diarrhea, abdominal pain, Crohn's disease, and *Clostridium difficile* infections (Borody and Campbell, 2011). There has been a recent increase in the use of FMTs as potential interventions to combat age-related diseases and even slow the aging process itself. Previous experiments using FMTs in mice have shown that transplanting young microbiomes into old animals decreases inflammation, improves intestinal barrier permeability, and restores visual function proteins (Parker et al., 2022). However, these changes, as well as the microbiome composition changes, returned to their prior state after 18 days, showing only a short-term effect of the reversals (Parker et al., 2022). Conversely, transferring the microbiome of an aged mouse to a young mouse negatively affects spatial learning and memory (D'Amato et al., 2020). In *Drosophila,* recently published work suggested a minor increase in longevity when flies are exposed to a young microbiome (Wesseltoft et al., 2024). In addition, flies exposed to a human microbiome showed increased lifespan and improved climbing ability with age, with the effects more pronounced in males (Ji et al., 2022).

In flies specifically, differences in individual microbes, environment, and diet can all impact the effects of the microbiome on health and longevity, and it can lead to conflicting results on the impacts of the microbiome on *Drosophila* health. For example, axenic flies, those raised without a microbiome, live longer when exposed to microbes early in life, but not late in life (Brummel et al., 2004). In addition, microbes can have different effects on fly lifespan on low-protein diets, suggesting microbiome by environment interactions may be prevalent (Keebaugh et al., 2019). Finally, interactions between bacterial species can directly influence lifespan and health variables in flies (Gould et al., 2018). Combined, these all suggest that more nuanced approaches are necessary for studying FMTs.

These environmental limitations are also coupled with other limitations in previous studies, such as using only male mice or only one genetic background in both mice and flies. As sex (Valeri and Endres, 2021) and genetic background (Cahana and Iraqi, 2020) can both shape the microbiome, there is a need for more comprehensive studies to understand the role of transplanting microbiomes on age-related health. Here, we use a novel method of microbiome transplants in multiple genetic backgrounds to determine their effects on age-related health and longevity, and to determine novel molecular changes that occur in response to microbiome changes.

## RESULTS

### Longevity and health

**Fig. 1.**
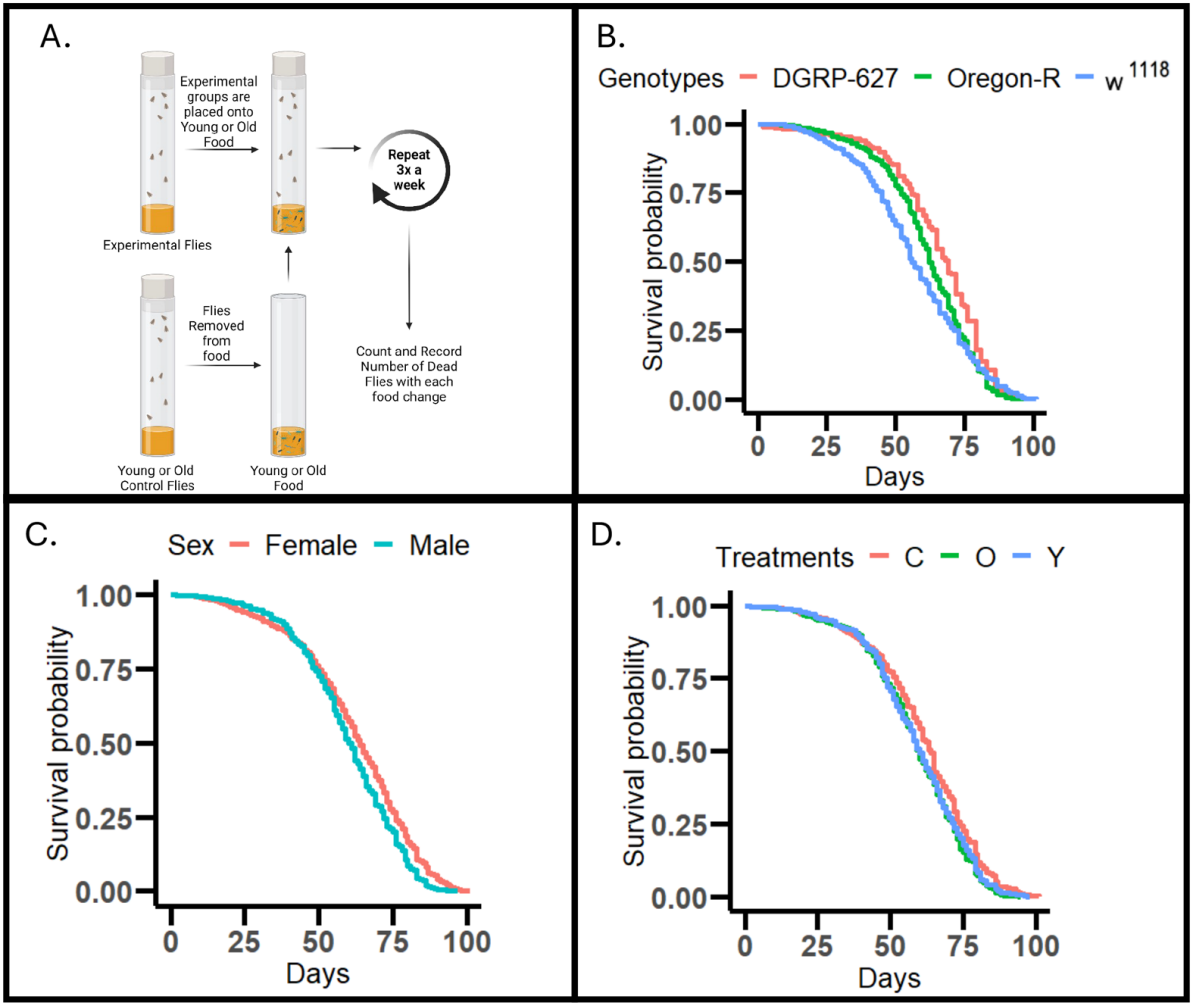
**Schematic (A) and longevity results (B-D).** (A) Overview of microbiome transplants in the experiment. Young or old flies were allowed to eat, regurgitate, and defecate on media for ∼48 h. Media was then fed to experimental flies. Figure made with BioRender. *N*=4600 individuals. Cox proportional hazards models used for all analysis. (B) Oregon-R (*P*=1.07e-10, hazard ratio=0.26) and *w^1118^* (*P*=1.19e15, hazard ratio=0.32) compared with DGRP-627. (C) Females (*P*≤2e-16, hazard ratio=0.28) compared to males (D) Young (*P*=8.55e-05, hazard ratio=0.20) and old (*P*=6.62e-07, hazard ratio=0.25) treated flies compared to control flies.

**Fig. 2.**
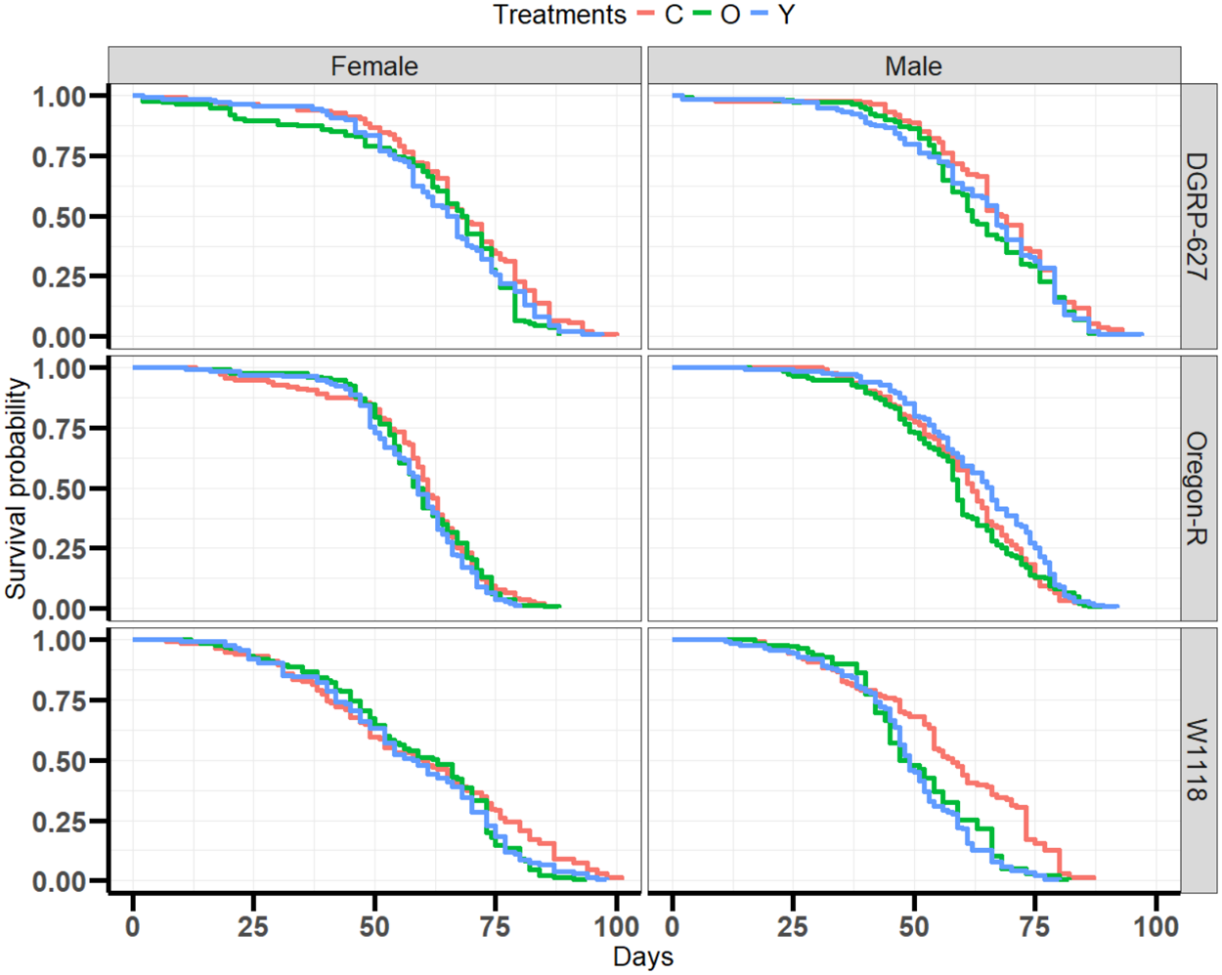
**Genotype and sex specific effects of microbiome transplants.** Control flies are in red while treatment flies are in green (old microbiome transplant) or blue (young microbiome transplant). *N*=3200 individual flies over two pooled experiments. Cox proportional hazards models used for analysis. All groups compared to control. Young (*P*=0.04391, hazard ratio=0.3270) and old (*P*=0.000851, hazard ratio=0.2446) DGRP-627 females. Young (*P*=0.239, hazard ratio=0.1449) and old (*P*=0.07, hazard ratio=0.2202) DGRP-627 males. Young (*P*=1.67e-08, hazard ratio=0.7286) and old (*P*=1.30e-06, hazard ratio=0.6181) *w^1118^* males. Young (*P*=0.0361, hazard ratio=1.2967) and Old (*P*=0.0235, hazard ratio=0.2859) *w^1118^* females. Young (*P*=0.0361, hazard ratio=0.2598) and old (*P*=0.0726, hazard ratio=0.2859) Oregon-R males. Young (*P*=0.0726, hazard ratio=0.21983) and old (*P*=0.5072, hazard ratio=0.08116) Oregon-R females.

We first investigated how our microbiome transplant affected longevity across the three genetic backgrounds, with flies fed either old or young microbiomes from middle age (Fig. 1A), and we looked at this across two replicate trials of which data was pooled for analysis. We found that the Oregon-R (*P*=1.07×10^−10^, hazard ratio=0.26) and *w^1118^* (*P*=1.19×10^−15^, hazard ratio=0.32) genotypes were shorter lived than DGRP-627 (Fig. 1B), and expectedly, male flies were shorter lived than the females (Fig. 1C, *P*≤2×10^−16^, hazard ratio=0.28). Both the young (*P*=8.55×10^−05^, hazard ratio=0.20) and old (*P*=6.62×10^−7^, hazard ratio=0.25) treated flies were significantly shorter lived than the control flies with young and old exposed flies having similar survival curves (Fig. 1D). However, the results of microbiome treatment were genotype and sex specific (Fig. 2), with Oregon-R males having increased lifespan on the young treatment compared to control flies (*P*=0.0371, hazard ratio=−0.26).

We then looked at the flies’ health when exposed to the different microbiome treatments. When pooling results from two replicates, we found, unsurprisingly, that climbing ability declined with age and was genotype specific (*P*≤2×10^−16^ for both). Old male flies performed significantly worse than females (*P*=0.000114, Fig. S2). However, we found no effect of treatment on climbing ability. For our heat stress assay, we found minor effects of treatment on survival at 38°C. DGRP-627 males exposed to the old treatment were significantly shorter lived (*P*=0.013, hazard ratio=0.46) compared to the control treatment, and DGRP-627 females on the young treatment were significantly shorter lived (*P*=0.0326, hazard ratio=0.38) than flies on the control treatment as well (Fig. S3), with no differences in any other genotype. When pooling our two oxidative stress assay replicates, male (*P*=0.0143, hazard ratio=0.44) and female (*P*=6.88×10^−5^, hazard ratio=0.78) DGRP-627 flies on the old treatment had shorter lifespans compared to the control treatment when exposed to hydrogen peroxide (Fig. S4). *w^1118^* male (*P*=0.0425, hazard ratio=−0.34) and female (*P*=0.00286, hazard ratio=−0.50) flies on the young treatment were significantly longer lived than the control treated flies under oxidative stress. When combining all treatments and genotypes, the old donor-treated flies were significantly shorter lived (*P*=0.00376, hazard ratio=0.21) than the other treatments.

Lastly for health, we looked at the effects of our microbiome treatments on gut integrity of the flies using a Smurf assay. Our Smurf assay led to a large number of deaths, potentially due to some toxicity of the blue dye, so in addition to measuring the number of smurfs, we also treated the data as a stress-response assay. Overall, only the DGRP-627 flies on the young treatment survived the food better than the control treatment (*P*=0.00967, hazard ratio=−1.07, Fig. S5A). No significant differences were seen between the treatments (χ-squared=0.32, *P*=0.85, Fig. S5B) nor between the sexes (χ-squared=0.2, *P*=0.66) in the number of smurfs observed, though there was a genotype effect where *w^1118^* flies were more likely to be smurfs (χ-squared=14.75, *P*=0.0006; Fig. S5D).

### Metabolomics

**Fig. 3.**
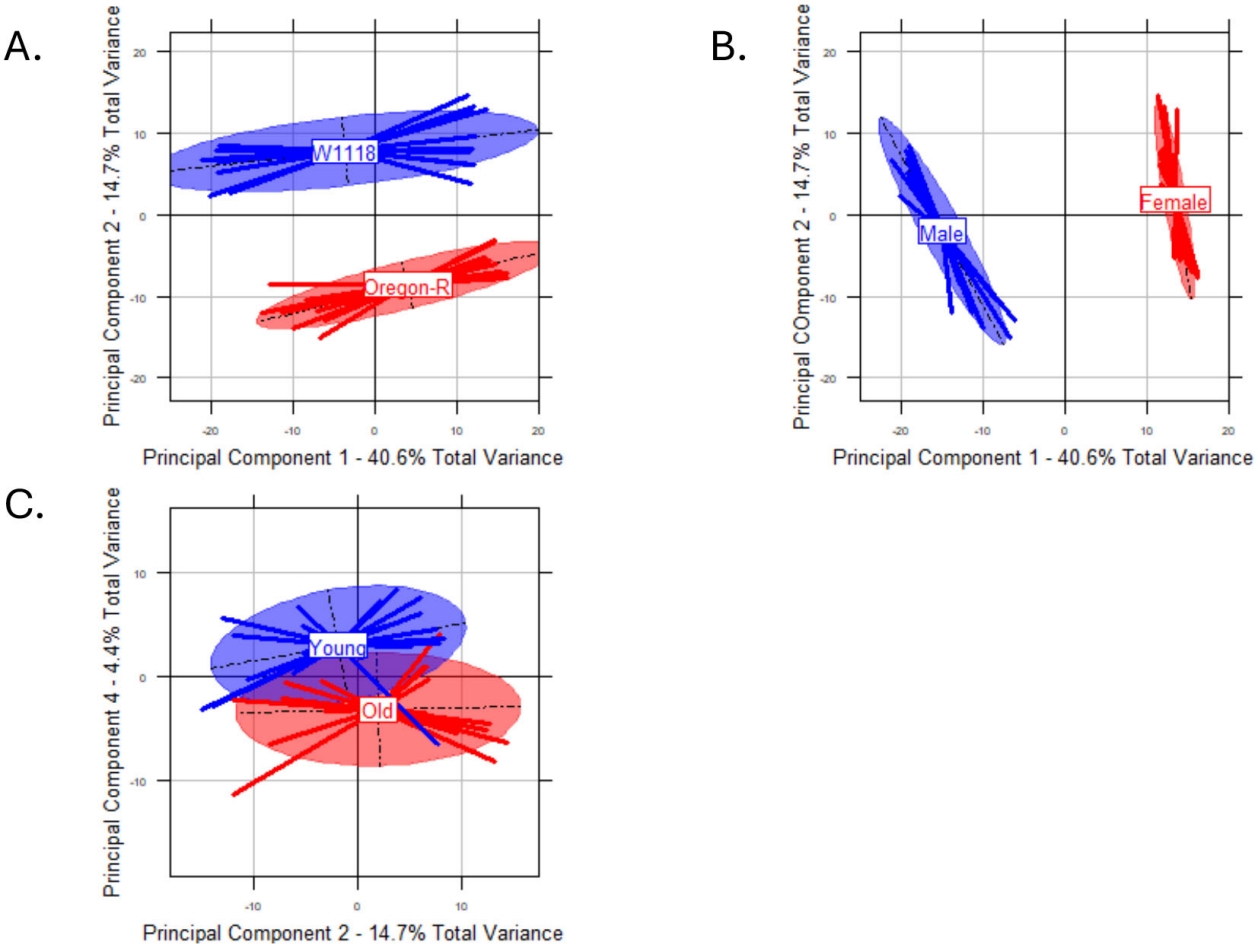
**Principal components analysis of metabolomics data.** (A) PC1 and PC2 highlighting the effects of genotype. (B) PC1 and PC2 highlighting the effects of sex. (C) PC2 and PC4 highlighting the effects of treatment. *N*=3-4 replicates per group. A, genotype-PC2 *P*≤2.2e-16. B, sex-PC1 *P*≤2.2e-16. C, treatment-PC4 *P*=1.77e-05. All analyzed with linear model.

Our metabolomics analysis consisted of 1021 initial metabolites across 38 samples (Table S1). After removal of missing values (see Materials and Methods), 669 metabolites remained. Based on our linear model (Table 1) and PCA analysis (Fig. 3), sex and genotype explained most of the variation in the metabolome, with only minor effects of treatment. Our metabolic pathway enrichment analysis found two pathways associated with genotype: itaconate degradation (*P*=0.03482) and methyl-coenzyme M reduction to methane (*P*=0.04258). One metabolic pathway was associated with sex: glycogen degradation I (*P*=0.04465). Acyl carrier protein metabolism (*P*=0.01837) and coenzyme A biosynthesis (*P*=0.02952) were associated with treatment. In our PCA analysis (Fig. 3), PC1, representing 40.57% of total variance, was significantly associated with sex (*P*≤2.2e-16), while PC2 (14.7% of the variance) was significantly associated with genotype (*P*≤2.2e-16). Microbiome treatment was associated with PC4 (*P*=1.77e-05).

**Table 1.** Table of total significant metabolites after false discovery function

Linear model	Number of significant metabolites	Percentage of significant metabolites
Genotype	307	45.9
Sex	443	64.7
Treatment	54	8.07
Genotype interaction	333	49.8
Sex interaction	403	60.2
Treatment interaction	30	4.5
Genotype and treatment interaction	84	12.6
Sex and treatment interaction	1	0.15
Genotype, sex, and treatment interaction	5	0.75

The number and percent of total metabolites that were significant after applying a false discovery function to the various different linear model results. Interaction models were combined models of genotype, sex, and treatment run together, and then separated after the model was produced.

### Metagenomics

Our metagenomics dataset consisted of 141,500 sequences per sample on average across 101 samples. Raw data is uploaded to NCBI GenBank, accession number: PRJNA1104669. Total sequences were reduced to 11,724.18 on average per sample after quality control and processing (see Materials and Methods). Nineteen total taxa were identified, including 16 genera and 10 species. The *Wolbachia* genus made up 75.13% of total matched sequences. Changes in overall *Wolbachia* concentrations varied across sex, genotype, and age (Table 2). Female flies had higher abundance of *Wolbachia* (*P*=3.15×10^−9^, estimate=−0.067, Fig. S6), and DGRP-627 flies had higher *Wolbachia* levels than both Oregon-R (*P*=1.84×10^−78^, estimate=−0.26) and *w^1118^* (*P*=7.39×10^−86^, estimate=−0.265). Young microbiome treated flies had a higher concentration of *Wolbachia* than old microbiome treated flies (*P*=0.0459, estimate=−0.03).

**Table 2.** Table of *Wolbachia* composition change across age

Percent abundance *Wolbachia*	Baseline	Control
Male	78.89	78.27
Female	81.91	71.52
*w^1118^*	74.94	54.89
DGRP-627	86.67	84.36
Oregon-R	79.04	86.14

**Fig. 4.**
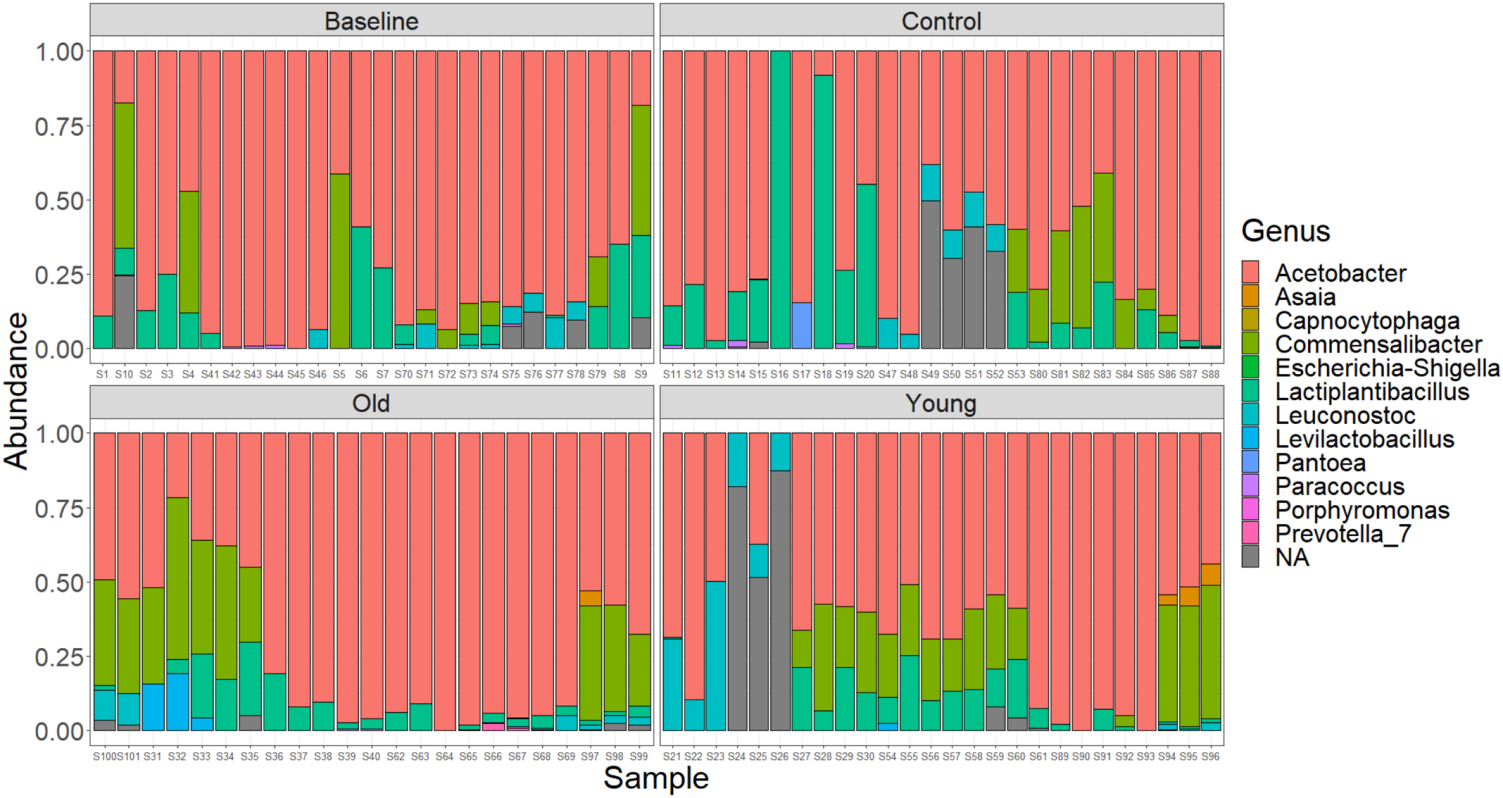
**Metagenomics results for baseline, control, young treated, and old treated flies.** Relative abundance is plotted for each sample. *N*=3-4 per group. One-way ANOVA, males: *P*=0.045, females: *P*=0.005 *w^1118^*: *P*=.0002 DGRP-627: *P*=0.027 Oregon-R: *P*=0.001.

**Fig. 5.**
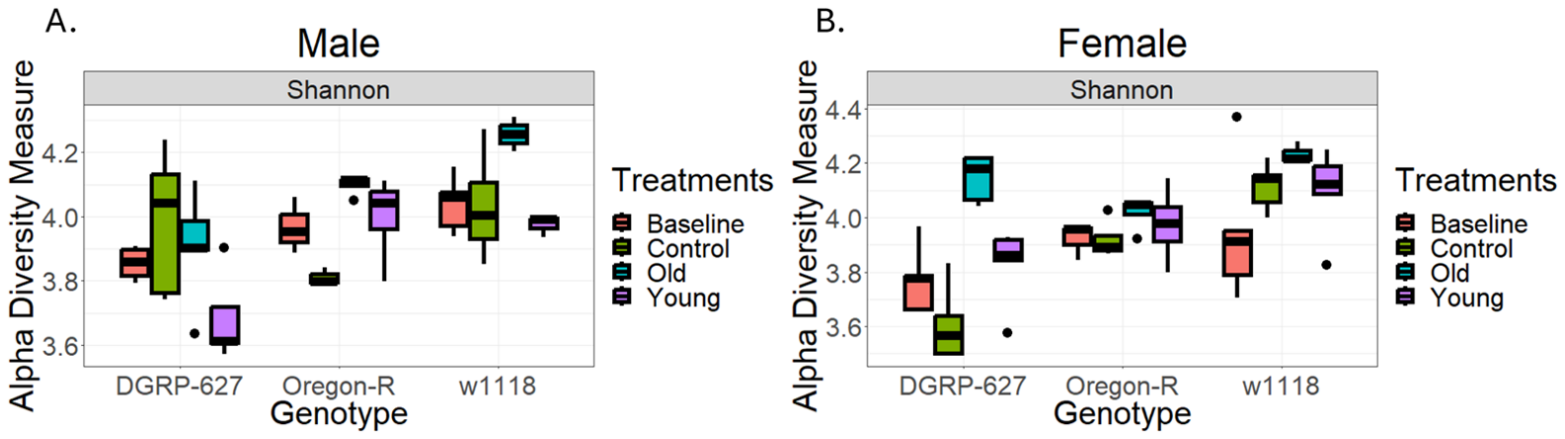
**Shannon Index alpha diversity levels for males (A) and females (B).** *N*=3-4 per group. One-way ANOVA, males: *P*=0.257. Females: *P*=0.053. *w^1118^*: *P*=0.02 DGRP-627: *P*=0.036 Oregon-R: *P*=0.001.

**Fig. 6.**
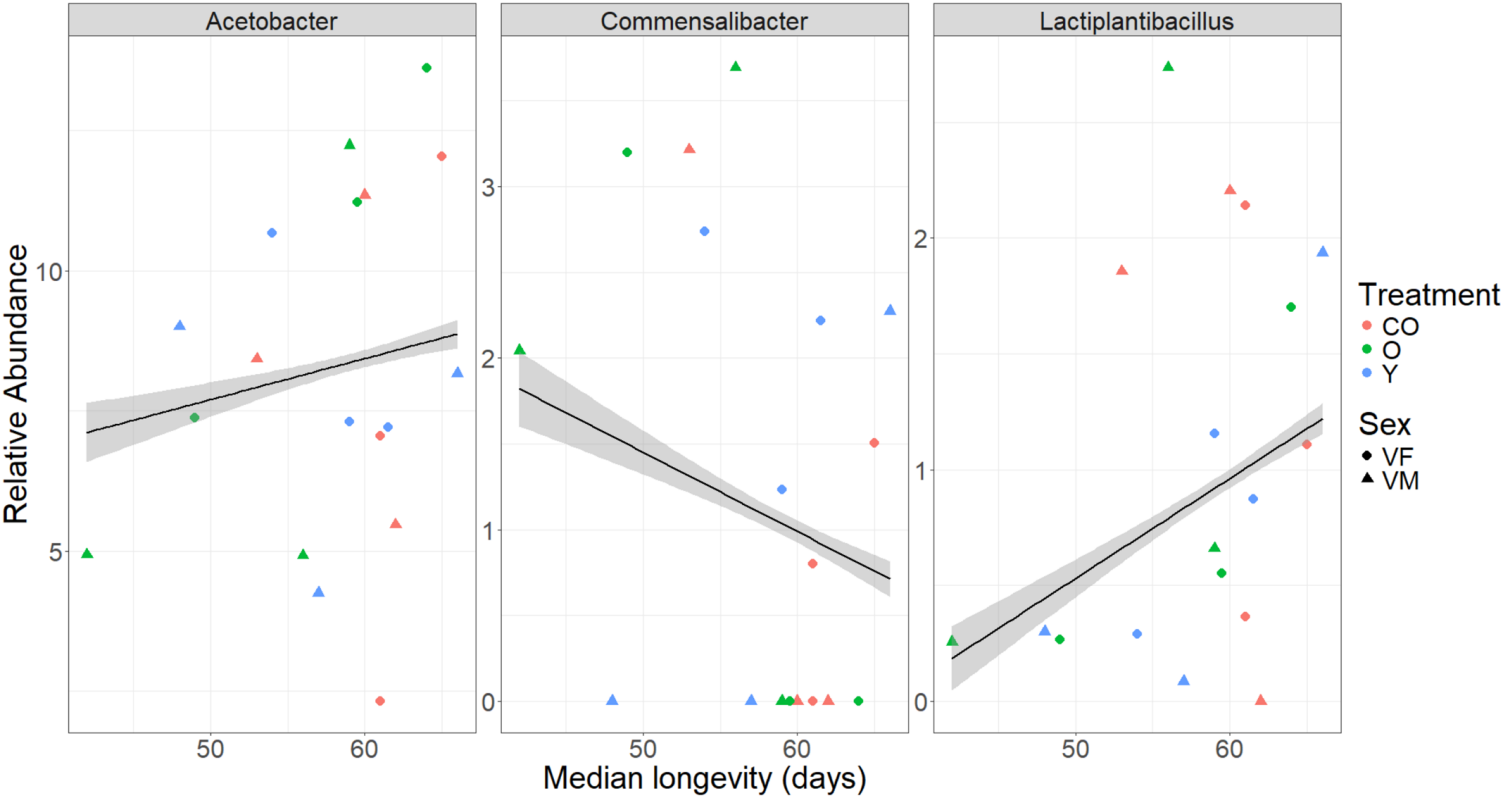
**Relative association of three genera of bacterial relative abundance with median longevity.** Control flies are in red while treatment flies are in green (old microbiome transplant) or blue (young microbiome transplant). Males (VM) are shown with triangles and females (VF) with diamonds. *N*=3-4 per group. Spearman's correlation rank test with longevity, Acetobacter: *P*=0.3952, rho=−0.00196. Commensalibacter: *P*=0.003163, rho=−0.0923. Lactiplantibacillus: *P*=1.056e-11, rho=0.171.

After removing *Wolbachia* sequences, the *Acetobacter* genus made up the largest component of the microbiome across all treatments (Fig. 4). Alpha diversity differed across treatments in males and females (*P*=0.045, *P*=0.005, respectively, Fig. 5A,B). All three genotypes also saw significant differences between the treatment groups (*w^1118^*: *P*=0.002, DGRP-627: *P*=0.027, Oregon-R: *P*=0.001). Overall, alpha diversity was increased for flies exposed to either the young or old treatment compared to baseline and control exposed flies. No significant differences were observed for the treatments for beta diversity (Fig. S7). No significant differences in bacterial load were found across groups (*P*=0.283). We also found significant changes with age in the microbiome (comparing baseline to control flies), though these changes varied by genetic background (Fig. S8). In addition, relative abundance levels of *Commensailbacter* were negatively associated with sex/genotype/treatment median longevity (Spearmon rho=−0.08, *P*=0.003), while abundances of *Lactiplantibacillus* were positively correlated with median longevity (Spearman rho=0.19, *P*=1.06×10^−11^, Fig. 6). There was no association between *Acetobacter* abundance levels and median longevity across the groups (Spearmon rho=−0.023, *P*=0.40).

**Fig. 7.**
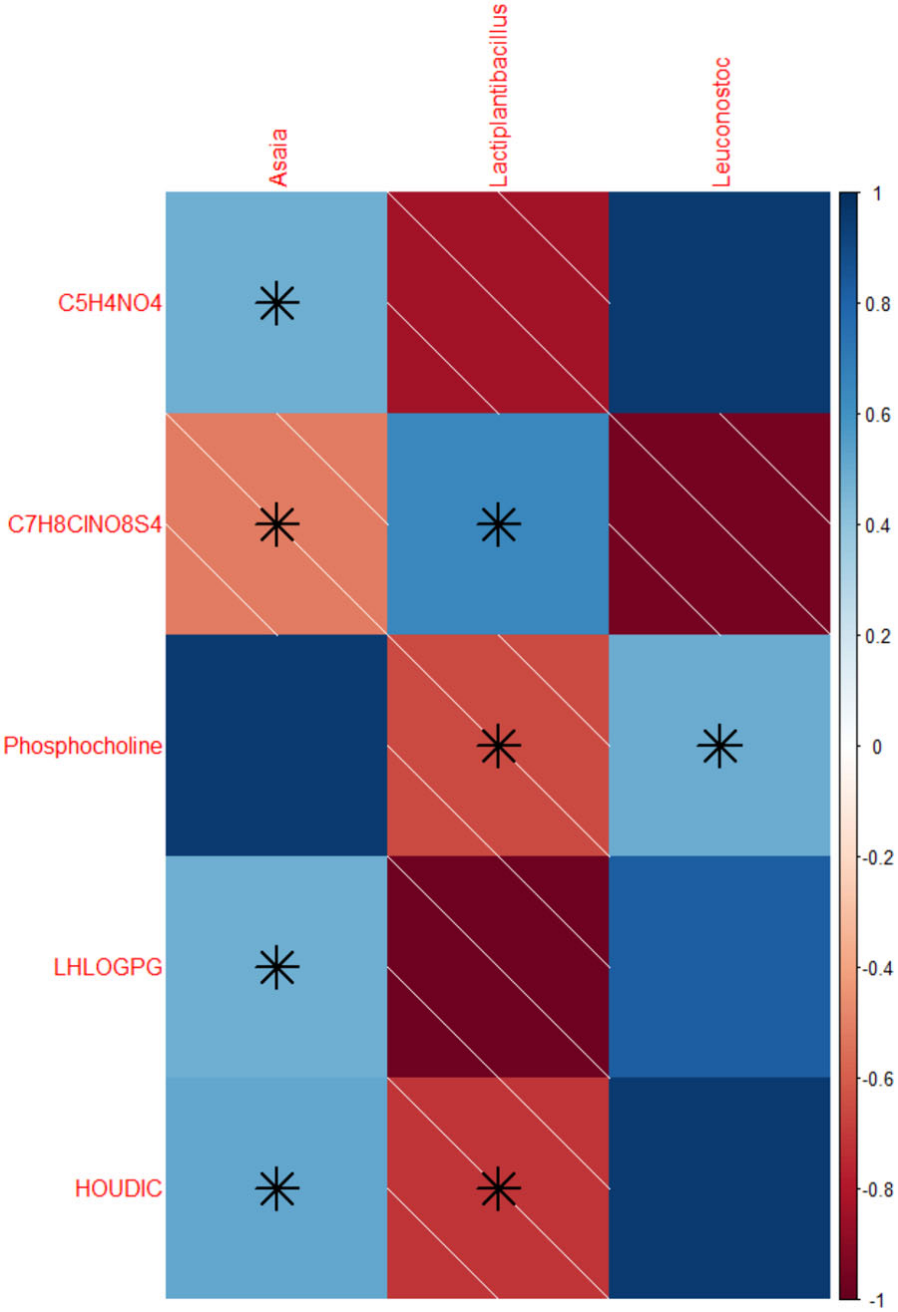
**Correlation of relative abundance of individual metabolites with relative abundance of microbiome genera.** Solid blue indicates a positive correlation while red with lines indicates a negative correlation. Boxes with stars were significant correlations, *P*<0.05. LHLOGPG is an abbreviation for 1-(6-[5]-ladderane-hexanoyl)-2-(8-[3]-ladderane-octanyl)-sn-glycero-3-phospho-(1′-sn-glycerol). HOUDIC is an abbreviation for 5-Hydroxy-2-oxo-4 -ureido-2,5-dihydro-1H-imidazole-5-carboxylate. *N*=3-4 per group. Spearman's correlation rank test, C5H4NO4 and Asaia: *P*=0.225, Spearman's rho=0.48. C5H4NO4 and Lactiplantibacillus: *P*=0.0154, Spearman's rho=−0.833. C5H4NO4 and Leuconostoc: *P*=0.000275, Spearman's rho=0.952. C7H8ClNO8S4 and Asaia: *P*=0.192, Spearman's rho=−0.514. C7H8ClNO8S4 and Lactiplantibacillus: *P*=0.0962, Spearman's rho=0.643. C7H8ClNO8S4 and Leuconostoc: *P*=0.000275, Spearman's rho=−0.952. Phosphocholine and Asaia: *P*=0.000165, Spearman's rho=0.959. Phosphocholine and Lactiplantibacillus: *P*=0.0781, Spearman's rho=−0.65. Phosphocholine and Leuconostoc: *P*=0.212, Spearman's rho=0.495. LHLOGPG and Asaia: *P*=0.225, Spearman's rho=0.483. LHLOGPG and Lactiplantibacillus: *P*=0.000397, Spearman's rho=−0.976. LHLOGPG and Leuconostoc: *P*=0.011, Spearman's rho=0.829. HOUDIC and Asaia: *P*=0.192, Spearman's rho=0.514. HOUDIC and Lactiplantibacillus: *P*=0.0576, Spearman's rho=−0.714. HOUDIC and Leuconostoc: *P*=0.000275, Spearman's rho=0.952.

Lastly, we are interested in understanding which metabolites were correlated with individual taxa of bacteria (Fig. 7). We found significant correlation across the two datasets, including *Leuconostoc* with C_5_H_4_NO_4_ (*P*=3.44e-5, estimate=0.976), likely uric acid, *Lactiplantibacillus* with 1-(6-[5]-ladderane-hexanoyl)-2-(8-[3]-ladderane-octanyl)-sn-glycero-3-phospho-(1’-sn-glycerol) (*P*=6.54e-05, estimate=−0.97), *Asaia* with phosphocholine (*P*=1.65×10^−4^, estimate=0.959), *Leuconostoc* with C_7_H_8_ClNO_8_S_4_ (*P*=9.16×10^−4^, estimate=−0.927), and *Leuconostoc* with 5-Hydroxy-2-oxo-4-ureido-2,5-dihydro-1H-imidazole-5-carboxylate (*P*=9.16×10^−4^, estimate=0.927).

## DISCUSSION

Here, we attempted to determine the effect of microbiome transplants on longevity and health in *D. melanogaster*. In general, we found microbiome transplants decreased the lifespan of flies (Fig. 1D), with slightly different responses across the sexes and genotypes (Fig. 2); the effects of the microbiome transplants on health were varied but overall minor. Sex and genotype were much stronger influences on health and longevity across the experiment.

Our results, suggesting a negative effect of microbiome transplants, support other studies’ findings that axenic flies live longer than conventionally raised flies (Lee et al., 2019; Shukla et al., 2021), and similar to our results where microbiome transplants were started in mid-life, axenic flies exposed to microbes late in life, but not early, were shorter lived (Brummel et al., 2004). While all our flies had a microbiome, the control flies had no ‘extra’ microbes in their diet. Therefore, lower levels of overall microbial composition may be beneficial for the health and survival of flies. Our results contrast a recent study also completing a microbial transplant in flies, which found that flies with a young microbiome were longer lived than old or control flies (Wesseltoft et al., 2024). This study used a very different method to feed the flies, using concentrated microbiomes with little other nutrition, and their overall lifespans are significantly shorter than would be expected in conventional housing. Both studies showed minor overall effects (either positive or negative) of the microbiome on longevity, suggesting microbial composition may not be a large contributor to variation in longevity, at least in *Drosophila*.

While our results were overall fairly neutral, we did find a significant increase in lifespan of young-exposed Oregon-R male flies compared to old-exposed flies that was repeatable across our two trials (Fig. 2). This is interesting, as it suggests there may be a specific microbe (or microbial composition) that is found in young Oregon-R male flies that does confer beneficial effects in late ages. In addition, our treatments started in midlife, when any translational intervention in humans would be more likely to be started. Often interventions begin in mid- or late-life fail to have significant impacts on longevity and late age health (Gonzalez-Freire et al., 2020), though this is not always the case (Harrison et al., 2009; Rana et al., 2017). Our Oregon-R results suggest there may be some potential that microbiome transplants, for specific genetic backgrounds, that can influence aging. Future studies are needed to look at the effects of Oregon-R microbiomes transplanted to other genetic backgrounds, and the overall results suggest we need to look at a wider range of genotypes, potentially with a cohort like the *Drosophila* Genetics Reference Panel (Mackay et al., 2012), to find responsive and non-responsive genotypes.

Overall, we found few effects of neuromuscular function changes in response to microbiome transplants. This is in contrast to prior studies which found that exposing axenic flies to fecal material improved climbing performance (Ji et al., 2022), and in another study, flies exposed to a young microbiome had better climbing ability than control flies (Wesseltoft et al., 2024). This difference, however, could be driven by genotypic differences and differences in microbiome delivery methods, as we found little to no effects of microbiome transplants on our stress assays, more similar to results from axenic flies. Previous reports found axenic and conventionally raised *Drosophila* show no difference in heat tolerance (Jaramillo and Castañeda, 2021), just as we found no effect of young and old microbiomes (Fig. S3). Interestingly, previous reports have found axenic flies performed significantly better when exposed to oxidative stresses than conventionally raised flies (Shukla et al., 2021). We found genotype specific effects in response to oxidative stress, where the *w^1118^* genotype was longer lived on the young treatment, but the other two genotypes were not. Combined, it appears the effects of microbiome transplants on health are still controversial, and most likely sex and genotype specific. Future studies are needed to incorporate more genetic backgrounds to understand the real effect of microbiome transplants on health and longevity in *Drosophila*.

It is important to consider that while we did not assess whether there would be any differences in effectiveness of the FMTs on different *Drosophila* diets, there have been several previous studies that have suggested significant microbe-by-diet interactions. For example, specific bacteria can cause negative health outcomes for flies on high nutrition diets and positive health outcomes on nutrient poor diets (Keebaugh et al., 2019). This is potentially because microbes have previously been found to be able to promote the harvesting of amino acids from the diet, as well as being a direct supplement of protein to the flies (Yamada et al., 2015). Additionally, *Lactiplantibacillus plantarum* was found to be able to promote larval growth in *Drosophila* during conditions of nutrient scarcity, which could impact the overall health of flies on low nutritional diets (Storelli et al., 2011). In addition, bacteria themselves can change the nutrition of a diet, such as increasing the ratio of protein to carbohydrates in the diet (Lesperance and Broderick, 2020). Some of our negative results may have been driven by the use of our diet; though fairly standard, it is lower nutrient (2.5% yeast, 8.5% sugar) than many other experimental diets. Future studies are needed to look more specifically at the effects of FTMs on different diets.

Similar to our previous results (Hoffman et al., 2014), we found sex and genotype were significant contributors to variation in the metabolome and a minor effect of microbiome transplants on overall metabolomic profiles. Unfortunately, the majority of the significant metabolites in our dataset were unable to be annotated, which limits our ability to understand how microbiome changes affect organismal metabolomic profiles. Future studies should potentially use a more targeted metabolomic panel to determine individual metabolites associated with shifts in the microbiome.

The *Wolbachia* endosymbiont is a major factor when assessing the microbiome of *D. melanogaster* due to its ubiquity, potential impact on mating, and other phenotypic effects (Serga and Kozeretskaia, 2013). In our samples, approximately 75% of the total sample sequences belonged to the *Wolbachia* genus, lower than previously reported abundance proportions (Veneti et al., 2004; Erkosar et al., 2018) (Table 2), potentially again due to large genotype effects that can be seen with *Wolbachia* abundance (Veneti et al., 2004). We also found sex differences in overall *Wolbachia* loads which are often not reported in the literature (Fig. S6). Unrelated to this study, it would be interesting to understand the drivers of these sex differences in *Wolbachia* composition, as it may be driving some of the many sex differences observed in flies.

Microbiome composition was similar, but not exact, between our study and previous results (Han et al., 2017). In our flies, the majority of the microbiome was composed of bacteria from the *Acetobacter* genus, while the *Lactobacillus* genus was a smaller component (Fig. 4). However, previous reports found that *Lactobacillus* was the most common taxa in older flies (Han et al., 2017). In *H. sapiens*, the diversity of the microbiome generally increases until approximately 80 years of age (Brooks et al., 2023), and we see this trend across our study (Fig. 5 and Fig. S6). However, as with all the previous results, these were genotype dependent. In addition, we found an increase in the ‘unknown’ taxa in the old-exposed flies, which has not been commonly reported before. If this is a repeatable finding, it will be interesting to follow up to determine what these undescribed species are in the guts of older flies.

We found that alpha diversity was higher in the old-exposed flies as compared to the young-exposed flies, and both had higher alpha diversity than the control flies (Fig. 5). Previous studies have been incongruent with the effects of alpha diversity on health and lifespan, with some studies suggesting increases in alpha diversity with age (Badal et al., 2020), while others showed no real change with age (de la Cuesta-Zuluaga et al., 2019). In addition, across neurodegenerative diseases, some are associated with increases in diversity while others are associated with decreases (Li et al., 2022). Overall, it would appear the changes in gut microbiome diversity may not drive differences in health and longevity across species. We should note that while alpha diversity was different across the treatments, there was no difference in overall microbial load. Microbial load has been linked to a wide variety of physiological outcomes, including negative health effects in flies (Clark et al., 2015), no impacts on fly longevity (Ren et al., 2007), and beneficial effects on lifespan in low nutrient conditions (Keebaugh et al., 2018). These previous studies combined with our results suggest that more attention is necessary to elucidate whether observed health effects are due to changes in the bacterial load or just in the changes abundance of specific bacterial species. This is important to decipher as interactions between bacteria can cause effects on host survival (Gould et al., 2018). Therefore, when bacterial load studies are performed, we must ensure that the exact ratio of bacteria within the gut remains the same, and only the overall number of bacteria increase.

Many of the metabolites required for physiological metabolism in the body are produced by the microbiome, so direct connections and relationships between the microbiome and metabolome are important (Fig. 6). For example, uric acid levels were highest in our shortest-lived strain and positively correlated with *Leuconostoc* concentrations. Overabundance of *Leuconostoc* bacteria has previously been associated with decreased lifespan in mutant flies (Dantoft et al., 2016). The effects of uric acid on *Drosophila* longevity are still inconclusive, with some suggesting levels decline with age causing a negative effect on health (Massie et al., 1991). In contrast, others suggest increased uric acid can shorten lifespan under certain dietary conditions (Lang et al., 2019). Our results, combined with these previous studies, suggest there may be an interaction of uric acid and *Leuconostoc* bacteria concentrations, which may affect the overall lifespan of the flies. Future studies looking at these metabolite–microbiome interactions are necessary to fully understand the role of alterations in these interactions on the physiology of an individual. We must note that while these correlations are interesting, one major drawback for *Drosophila* is that both metabolomics and metagenomics are destructive processes. Obtaining both metagenomics and metabolomics data from the same fly is impossible due to destructive sampling, so correlations must be between flies of the same genotype, sex, and treatment, which reduces the power of our results.

### Caveats

While our study is one of the largest microbiome and aging studies in *Drosophila*, it is not without its limitations. We find potentially negative effects of microbiome transplants on *Drosophila* longevity and health, but our study may be limited by our experimental design. Potentially, not all microbes on the food are making it into the gut to colonize. However, this method of microbiome reconstitution, using media from previous flies, has been used previously to establish a microbiome in other studies (Chaplinska et al., 2016), and we find significant shifts in the microbiome composition after exposure to the different treatments suggesting that the transplants did work. Our metabolomic and metagenomic analysis used whole flies to look at the effects of our transplants. However, by looking at the whole organism we may have missed some smaller changes that occurred in the gut specifically. This might explain why we only had one responsive genetic background/sex (Oregon-R males) to our microbiome treatment, as the Oregon-R genotype may have had a specific microbe that caused the positive effect, as described earlier in the discussion. However, we do think that using the whole fly also allowed us to make some interesting findings about sex and genotype changes in *Wolbachia* that are often not reported. Lastly, while we had two trials of our longevity experiment that were pooled together, we still had quite noisy longevity curves when broken down by genotype and sex (Fig. 2), and for a couple of the groups, we saw mild separation of the longevity curves before treatment began. These could be driven by microbial differences between replicates, as well as small stochastic differences across replicates which can have significant effects on longevity curves (Hoffman et al., 2021). Future studies with more genotypes and replicates may help reduce the overall noise seen across longevity cohorts.

### Conclusions

We found a minor, negative effect of microbiome transplants on *Drosophila* lifespan, which is the opposite of our hypothesis. Our results, combined with those of other researchers, suggest that the effects of microbiome transplants in *Drosophila* are potentially minimal and genotype and sex specific, as well as dependent on the method of delivery. Combined with the transient nature of insect guts, we would conclude that *Drosophila* may not be an ideal model for understanding the translational potential of microbiome transplants for human biomedical health.

## MATERIALS AND METHODS

### Fly husbandry

*D. melanogaster* fly stocks (*w^1118^*, Oregon-R, and DGRP-627) were acquired from the Bloomington Stock Center. Flies were maintained on a 12:12 light:dark cycle at 25°C and 60-70% humidity and were fed a standard diet of 10 g/L agar, 85 g/L sugar, 60 g/L cornmeal, 25 g/L yeast, and 3 ml propionic acid. Male and female flies were collected as virgins in groups of 20 under light CO_2_ anesthesia shortly after eclosion.

### Microbiome transfer

Microbiome donor flies were placed on new food for 48-72 h. During this time, flies salivate, regurgitate, and defecate on the food, introducing their microbiome (Fig. 1A). Donor flies were either young (Y, less than 27 days of age) or old (O, over 41 days of age). Middle aged (∼30 days) recipient flies were randomized onto food with the microbiomes from the donor flies. Flies received transfers from either Y or O donors for the duration of their lives or were fed control food that was not exposed to any flies beforehand (C); we used a lifelong treatment to increase the effectiveness of the transplants as microbiomes in *Drosophila* have been shown to be quite transient (Broderick and Lemaitre, 2012). Food provided to flies came from the same genotype and sex of fly, e.g. *w^1118^* male recipients received food from *w^1118^* male donors. A second control was initially used in which vials were exposed to the environment of the donor flies for 48-72 h without any flies in the vial. This allowed any microbes from the air to settle on the food, and we could determine whether these environmental microbes had any effect on fly health and longevity. This additional group was combined with the standard control group for all results presented, as there was no difference between the two (log-rank *P*=0.30, Fig. S1).

### Longevity

Approximately 100 flies from each group (Y, O, C) were randomized onto their treatment at ∼30 days of age. Flies were transferred onto new media three times a week, and deaths were recorded at time of transfer. Transfers continued until all flies were dead. Two trials of the longevity assay were completed.

### Health assays

We first measured the neuromuscular function of the flies using a standard climbing assay (Gargano et al., 2005), as reduction in climbing ability with age is well described in flies (Rhodenizer et al., 2008). Climbing assays were performed the day that treatment began for the flies (∼30 days old), 3 weeks (∼50 days old) after onset of treatment, and 5 weeks (∼65 days old) after treatment started. Briefly, flies were placed in an empty vial, tapped to the bottom, and given 10 s to climb. Flies that climbed past a 5 cm mark in the 10 s were recorded. Two trials of the climbing assay were completed and pooled for analysis.

Resilience to stressors naturally declines over the lifespan of *D. melanogaster,* including both heat stress (Sørensen and Loeschcke, 2002) and oxidative stress (Parashar et al., 2008). Heat and oxidative stress assays were performed on new cohorts of flies that had been on the treatment 4 weeks (∼60 days old). For heat stress, flies were placed into an incubator set at 38°C and the number of dead flies was recorded every hour. For oxidative stress assay, flies were placed on food containing 90 ml H_2_O, 8.3 g agar, 5.6 g dextrose, 3 ml propionic acid, and 185.2 ml H_2_O_2_, modified from a previous study (Harrison et al., 2020). They were then placed into a standard incubator, and the number of dead flies was recorded every 4-8 h. One trial of the heat stress assay was conducted, and two trials of the oxidative stress assay were performed.

To measure intestinal integrity of *D. melanogaster*, we completed a smurf assay on microbiome treated flies (Rera et al., 2011). Middle aged flies were raised on their respective microbiome treatment (C, O, Y) for 3 weeks and then were given a standard *Drosophila* media that included 2 g/l of Brilliant Blue R 250. After 2 weeks of consuming the media blue dye mixture, the flies were examined under a dissecting microscope to determine to what extent they had become ‘smurf’ flies. Flies with blue coloring only within their guts, or none at all were classed as non-smurfs, and flies with blue coloring anywhere outside the gut were classed as smurfs.

### Metagenomics and metabolomics sample collection

For metabolomics, treatment flies were collected from two genotypes (*w^1118^* and Oregon-R) after 1 month of treatment. For metagenomics, flies were collected before the start of treatment as well as 1 month after treatment. Flies were anesthetized on ice and placed in groups of 10 whole flies into Eppendorf tubes. Tubes were then flash-frozen in liquid nitrogen and stored at −80°C until sample processing began.

### Metabolomics

Samples were run at the Proteomics Core at Augusta University. Each sample was homogenized with 1000 µl of 80% methanol at −20°C using 0.1–0.2 ml of zirconium oxide beads on a Blue Bullet Blender run at a speed of 8 for 3 min in a cold room. An internal standard was added to the tube at this time. The lysate was then incubated at −20°C for between 4 h and overnight to precipitate proteins. Samples were then vortexed for 30 s and centrifuged at 20,000 ***g*** for 15 min at 4°C. 900 µl of the supernatant was transferred to a new tube and dried completely with a SpeedVac without heat. Samples were then reconstituted by adding 100 µl of 50% methanol and vortexed for another 30 s. Samples were placed in an ice bath for 10 min and then centrifuged at 16,000 ***g*** for 10 min. This supernatant was then transferred to a glass vial, capped, and put into the sample loading tray.

A ^13^C-labeled internal standard was created by dissolving 1 µl of heavy phenylalanine in 1 ml of 80% methanol. 5 µl of the internal standard was injected into the LC-MS device. Untargeted analysis was completed using Agilent 1260 Infinity II LC coupled with an Agilent 6520 Quadrupole-Time of Flight (TOF)-MS system (Agilent Technologies). The ESI voltage was 3.8 kV, and the *m/z* scan range was 60–1000. The LC column consisted of a Waters Xbridge Amide column measuring 50 mm by 2.1 mm with a matching pre-column. Mobile Phase A was 5 mM ammonium acetate in 90% water and 10% acetonitrile with 0.2% acetic acid. Mobile Phase B was 5 mM ammonium acetate in 10% water and 90% acetonitrile with 0.2% acetic acid. The flow rate was 0.3 ml/min, the column temperature was 40°C, and the total run time was 20 min. The LC-TOF-MS data was extracted using Agilent MassHunter Qualitative Analysis (version B.08.00), Quantitative Analysis (version B.08.01), and Mass Profiler Professional (MPP, version B.13.00) software. The absolute intensity threshold for the LC-TOF-MS data extraction was 1000, and the mass accuracy limit was set to 10 ppm. The mass to charge ratio (m/z) was calculated for each hit to help determine identity. Missingness was assigned to peaks below 4500 counts per second. The resulting data was then exported to Excel for further analysis.

### Metagenomics

Sample extraction and processing were completed by Norgen. Briefly, flies were crushed into a powder using a mortar and pestle under liquid nitrogen. Lysis Buffer B was added to the powder, and the mixture was transferred into a 2 ml tube. DNA isolation was then performed using Norgen's Cell and Tissue DNA Isolation Kit (catalogue number 53100), using manufacturer guidelines. DNA quality checking (QC) was then performed using a NanoDrop 2000 (Thermo Fisher Scientific). 16S rRNA library preparation used Norgen's 16S V3-V4 Library Preparation Kit for Illumina (catalogue number 70410 and 70440) using manufacturer guidelines. Library QC was then generated using a Bio-Rad CFX Touch 96 PCR machine with the Kapa Library Quantification kit (catalogue number 07960140001) using manufacturer guidelines. The generated libraries were then pooled together and sequenced using the Illumina MiSeq platform with the MiSeq Reagent Nano Kit version 3 (600 cycles).

### Statistical analysis

All data was analyzed in R, version 4.2.1 (Team, 2010), unless otherwise stated. First, Cox proportional hazard models and log-rank tests were run with the survival package (Therneau and Lumley, 2015), looking at the effects of sex, genotype, microbiome treatments, and their interactions, and Kaplan–Meier curves were generated. All deaths that occurred prior to 32 days were censored to remove deaths not due to intervention. Similar methods were used for oxidative and heat-stress assays. For the climbing assay, linear regression models were run between the percent of climbing flies and age, sex, treatment, genotype, and their interactions. Smurf assay differences of sex, genotype, and treatment were determined by chi-squared tests.

For metabolomics data, raw mass spectrometry intensity data was first log10 transformed, and any resulting metabolites with more than 30% missing values were removed. The remaining metabolites were then centered and scaled. A linear regression model was run for each metabolite, looking at sex, genotype, treatment, and all their interactions. A Benjamini-Hochberg (Benjamini and Hochberg, 1995) false discovery rate at alpha=0.05 was applied to determine significant metabolites. Metabolites were then run through MetaboAnalyst 6.0 (Lu et al., 2022) for annotation of m/z peaks, and we then looked at individual metabolic pathways that were enriched for the different factors of interest based on significant metabolites found in our linear models. We lastly completed a principal components analysis (PCA) of the data using the ade4 package (Bougeard and Dray, 2018). Metabolites with more than 10% missing values were removed from the analysis, and remaining missing values were imputed using the metabolite median value.

Metagenomics data was assessed using the default settings of the dada2 pipeline, which produced ASV files from the raw fastq reads acquired from Norgen (Callahan et al., 2016). Briefly, forward and reverse fastq files were filtered and trimmed based on the quality profiles; then remaining sequences were combined with a calculated error rate to produce forward and reverse dada-class objects. The forward and reverse dada-class objects were then merged together, chimeras were removed, and taxonomy was assigned using the Silva v138 Species training set, and the resulting taxonomic data was converted into a phyloseq-class object for further analysis (Quast et al., 2013; McMurdie and Holmes, 2013; Yilmaz et al., 2014).

We first ran a statistical analysis of all data and then removed those sequences identified as *Wolbachia. Wolbachia*, an endosymbiont found throughout *Drosophila* cell types*,* is more abundant than other gut-dwelling species, making analyses difficult in their presence. We calculated the Shannon Index for alpha diversity, and richness plots were generated before statistical analysis using a PERMANOVA from the vegan package (Oksanen, 2010). Then beta diversity was assessed, and a permutation test for homogeneity of multivariate dispersions was performed between each treatment group (Oksanen, 2010). After this, the bacterial load of samples was calculated by determining the total count of microbes in each sample and differences were calculated using a one-way ANOVA. Next, ordination plots using the NMDS method with bray distances were generated for each sex and genotype separately (Bray and Curtis, 1957; Zhu and Yu, 2009). Sample counts were first transformed into relative and percent abundances, combining samples with the same taxa. We then generated abundance plots across groups (Wright, 2016; Pagès et al., 2024; Wickham, 2016; Wickham et al., 2019; Lahti, 2019; Oksanen, 2010; Kassambara, 2018; Dowle et al., 2019). We then ran a GLM with a negative binomial distribution on the count data for each genus across samples, looking at the effects of sex, genotype, and treatment. We then ran a Spearman’s rank correlation coefficient test on the most abundant bacterial genera and median longevity. Lastly, we calculated correlations between individual metabolites and microbiome abundance levels (Wei et al., 2017; Wickham and Wickham, 2017a,b).

## Supplementary Material

10.1242/biolopen.061745_sup1 Supplementary information

Table S1.
