# Supplementary material for: Microbiome transplants may not improve health and longevity in Drosophila melanogaster
Source: Biol Open. 2025 Jan 21;14(1):bio061745. doi: 10.1242/bio.061745 (PMC11789278; doi:10.1242/bio.061745)
Supplement: Supplementary information [file biolopen-14-061745-s1.pdf]

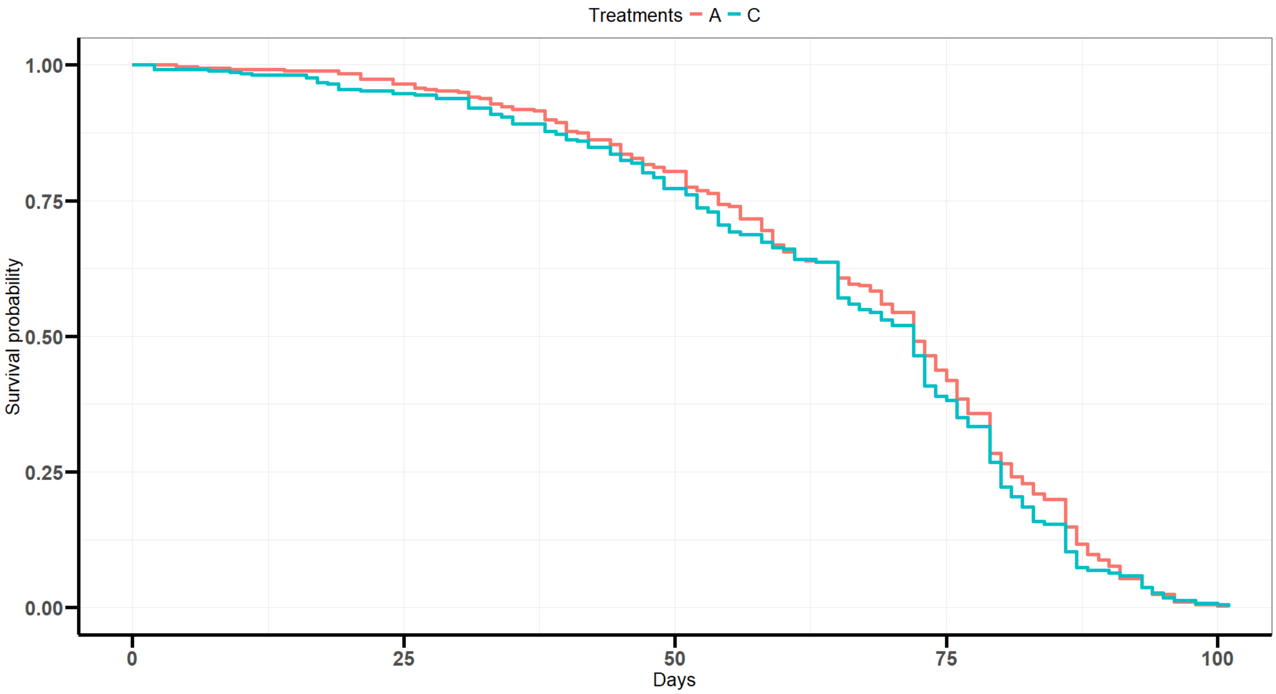

Fig. S1. Longevity curves of air exposed (labeled A) and control food (labeled C). Air food was exposed to the air for the same amount of time as media was exposed to flies for microbiome transfer. Control food was never exposed to any air (see methods).

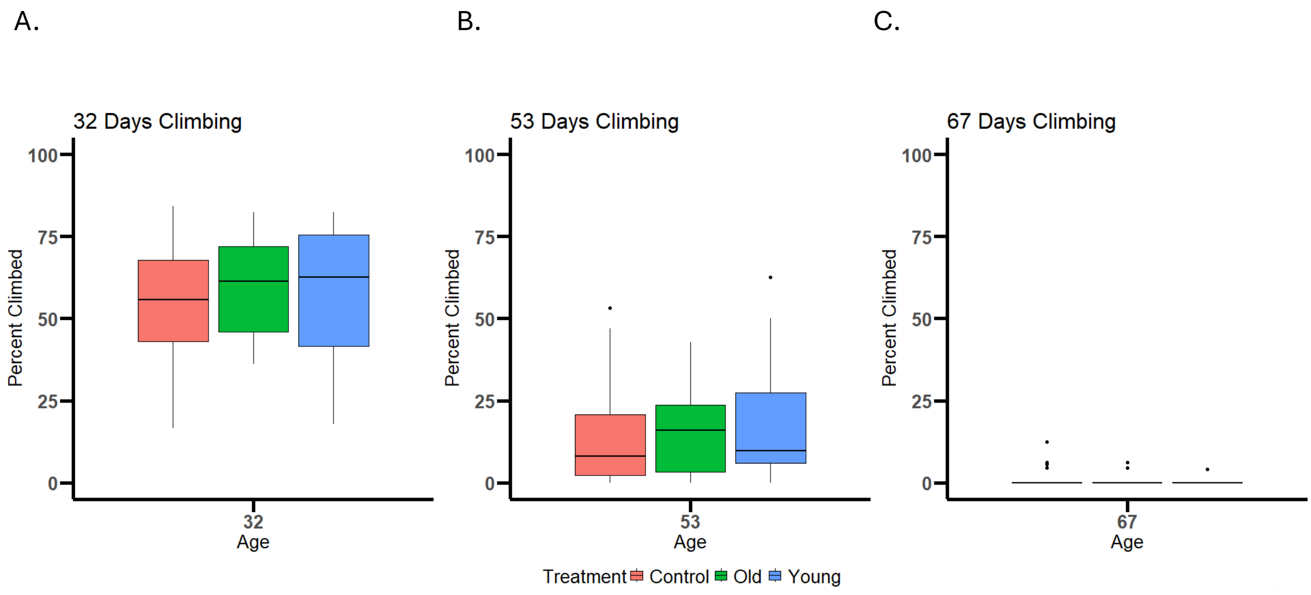

Fig. S2. Climbing assay results for control (red), young treated (blue), old treated (green) flies. A) Middle age before any treatments. B) After 3 weeks of treatment. C) After 5 weeks of treatment.

Biology Open • Supplementary information

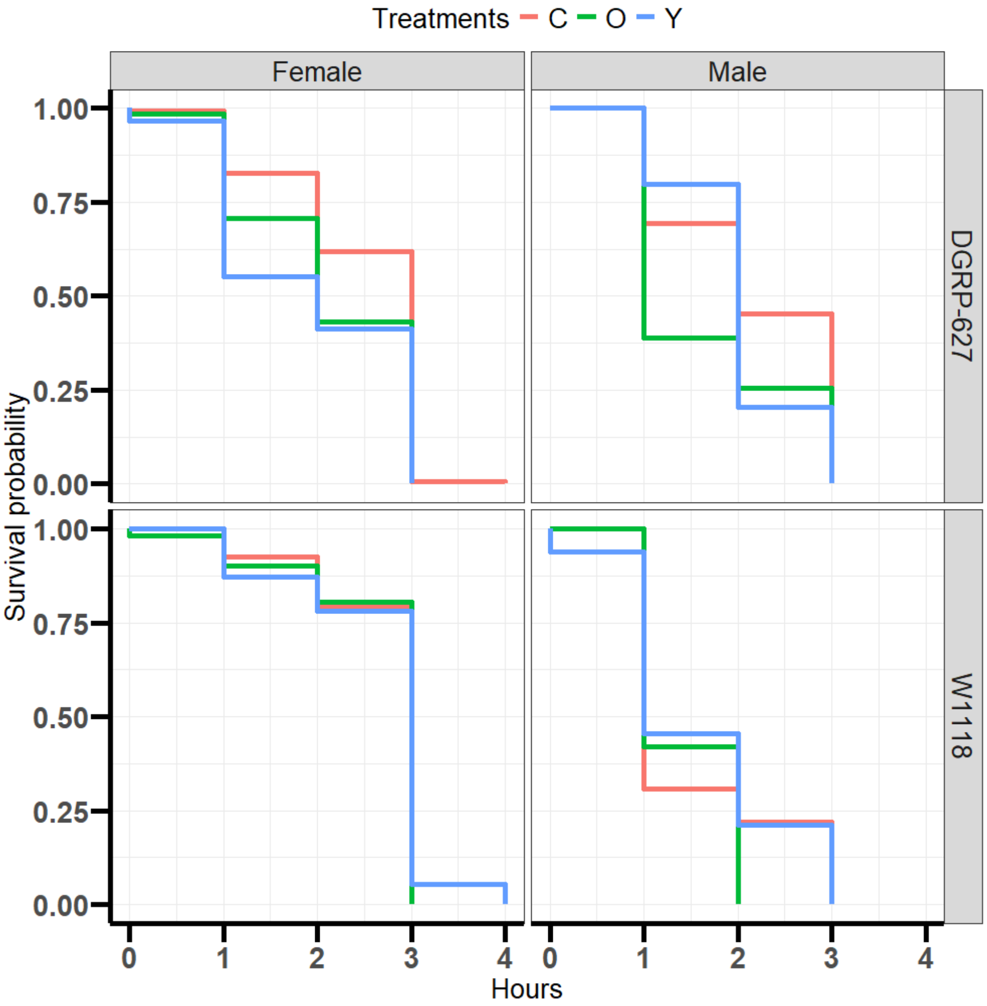

Fig. S3. Heat stress survival curves for control (red), young treated (blue), old treated (green) flies.

Biology Open • Supplementary information

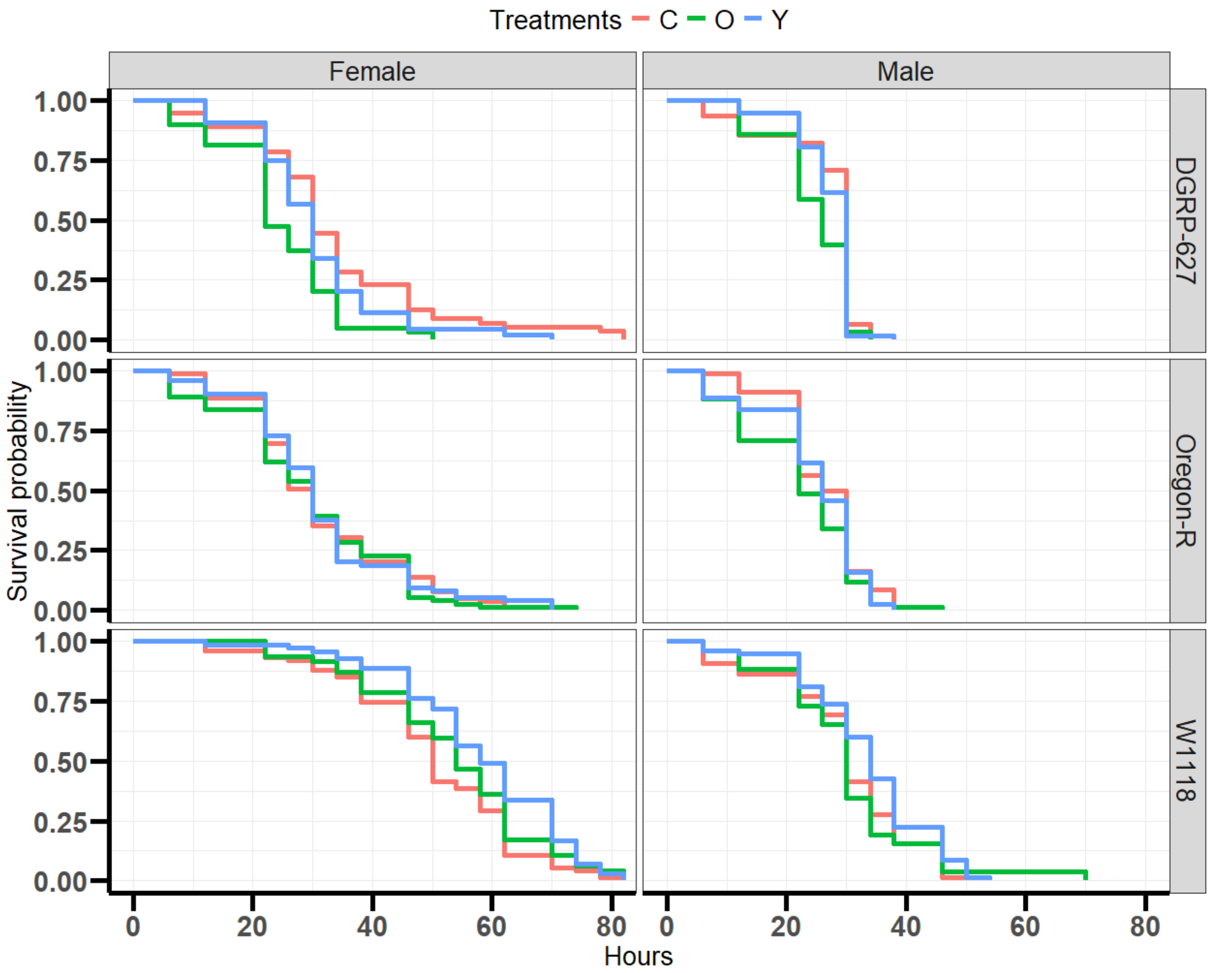

Fig. S4. Oxidative stress survival curves for control (red), young treated (blue), old treated (green) flies.

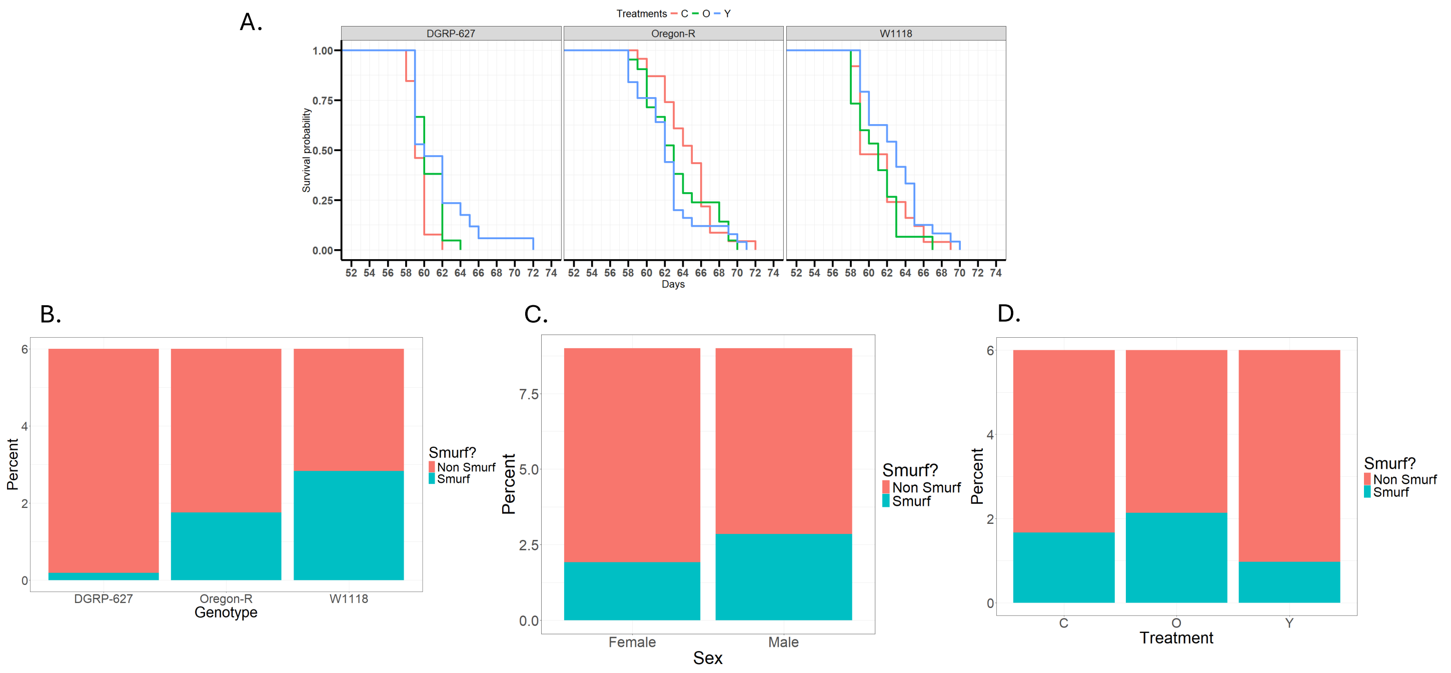

Fig. S5. Smurf assay results. A) Survival curves for flies placed on blue food dye: control (red), young treated (blue), and old treated (green) flies. B) number of smurfs by genotype. C) Number of smurfs by sex. D) Number of smurfs by treatment.

Biology Open • Supplementary information

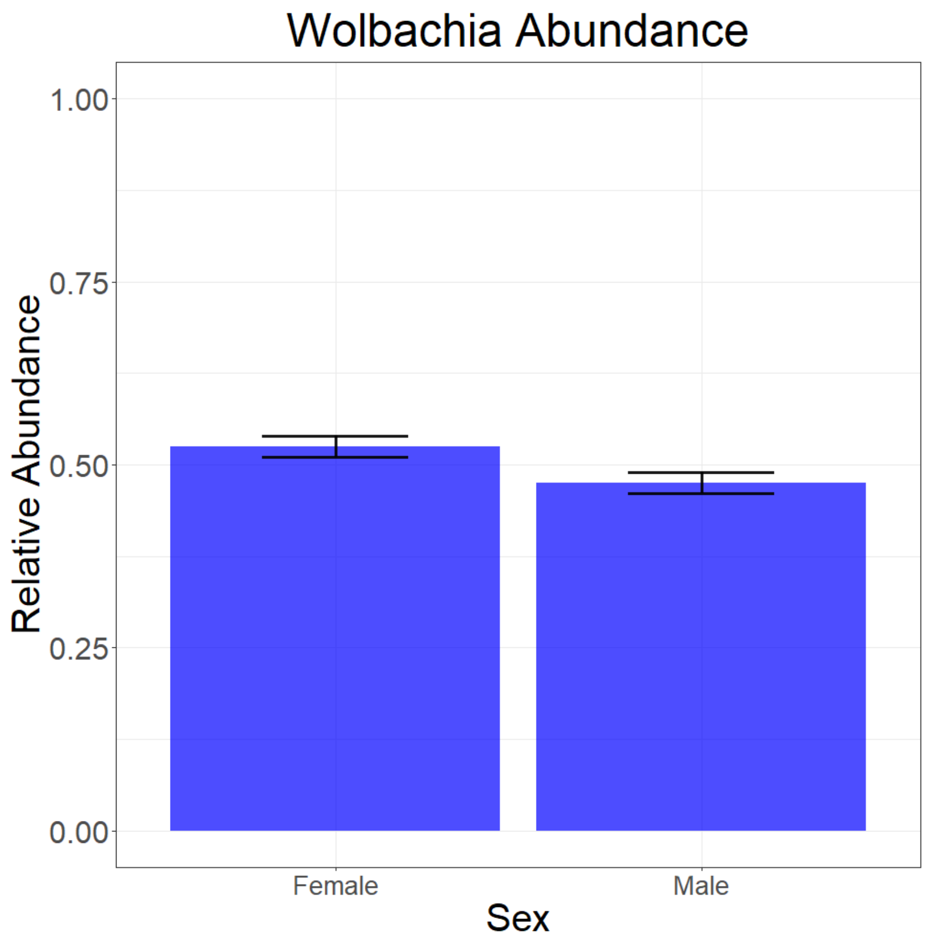

Fig. S6. Sex differences in relative abundance of Wolbachia

Biology Open • Supplementary information

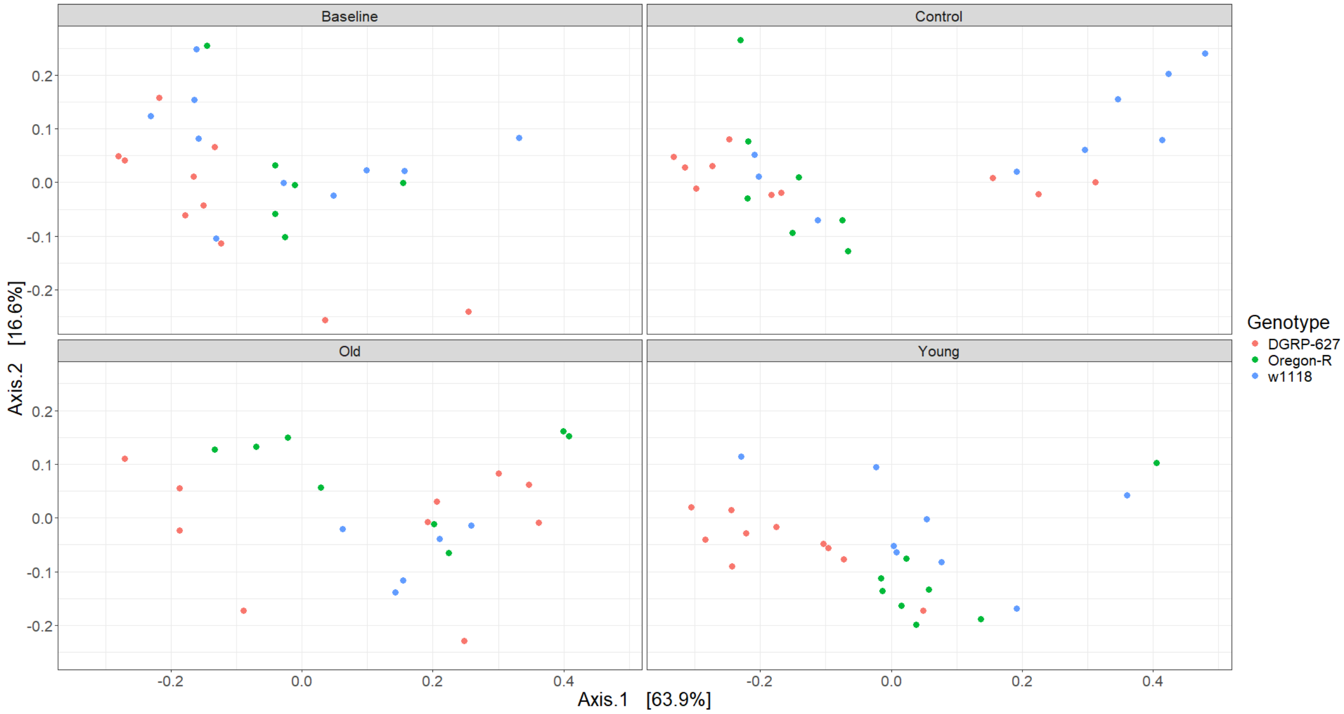

Fig. S7. Beta diversity across all four treatments.

Biology Open • Supplementary information

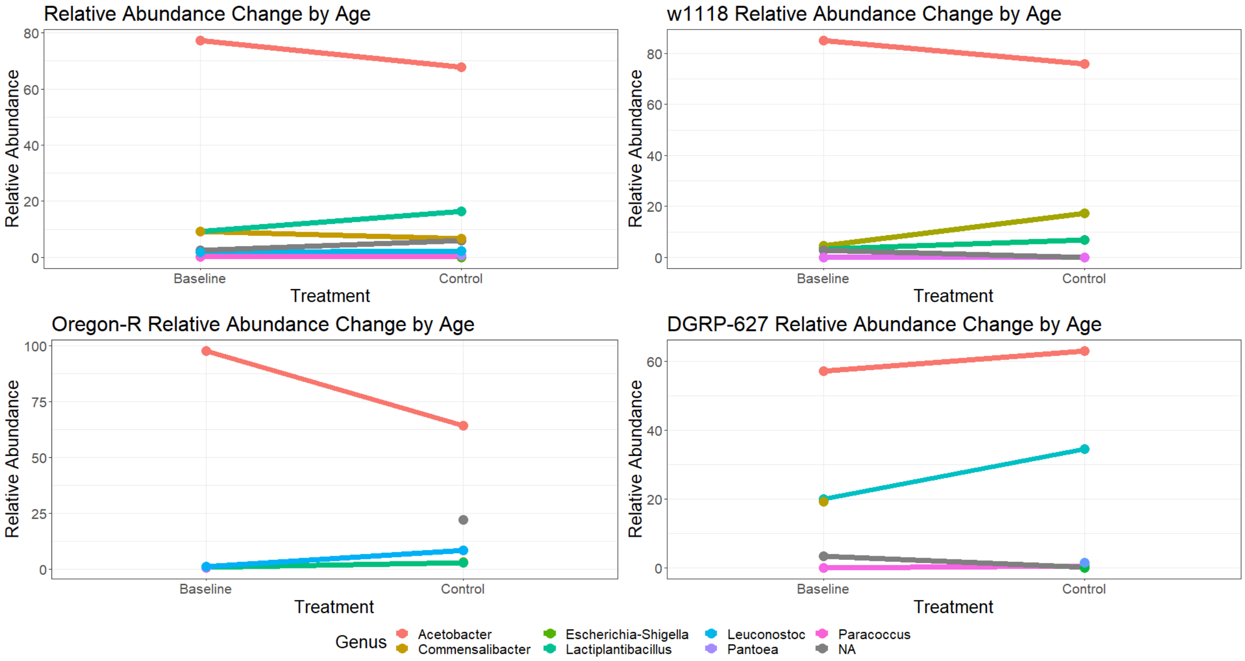

Fig. S8. Changes with age (baseline to control values) for different genera of microbiota.

Table S1.

Available for download at
https://journals.biologists.com/bio/article-lookup/doi/10.1242/bio.061745#supplementary-data

Biology Open • Supplementary information
